# Is It Reliable to Use Common Molecular Docking Methods for Comparing the Binding Affinities of Enantiomer Pairs for Their Protein Target?

**DOI:** 10.3390/ijms17040525

**Published:** 2016-04-20

**Authors:** David Ramírez, Julio Caballero

**Affiliations:** Centro de Bioinformática y Simulación Molecular (CBSM), Universidad de Talca. 2 Norte 685, Casilla 721, Talca, Chile; damach.david@gmail.com

**Keywords:** molecular docking, modeling of enantiomers, prediction capability, docking accuracy, docking scoring, binding affinities

## Abstract

Molecular docking is a computational chemistry method which has become essential for the rational drug design process. In this context, it has had great impact as a successful tool for the study of ligand–receptor interaction modes, and for the exploration of large chemical datasets through virtual screening experiments. Despite their unquestionable merits, docking methods are not reliable for predicting binding energies due to the simple scoring functions they use. However, comparisons between two or three complexes using the predicted binding energies as a criterion are commonly found in the literature. In the present work we tested how wise is it to trust the docking energies when two complexes between a target protein and enantiomer pairs are compared. For this purpose, a ligand library composed by 141 enantiomeric pairs was used, including compounds with biological activities reported against seven protein targets. Docking results using the software Glide (considering extra precision (XP), standard precision (SP), and high-throughput virtual screening (HTVS) modes) and AutoDock Vina were compared with the reported biological activities using a classification scheme. Our test failed for all modes and targets, demonstrating that an accurate prediction when binding energies of enantiomers are compared using docking may be due to chance. We also compared pairs of compounds with different molecular weights and found the same results.

## 1. Introduction

Molecular docking has become a major computational method for the prediction of ligand–receptor interactions [1] and is an important and powerful tool for rational drug design [2]. Over the last few years the number of new molecular targets has increased due to the completion of the human genome project, as well as the protein and protein–ligand complex structures isolated by high-throughput protein purification [3] and solved by crystallography and nuclear magnetic resonance spectroscopy techniques [4,5]. At the same time, the improvement of computational techniques for studying interactions of ligands with the biological targets at the atomic scale have increased and developed.

**Figure 1:**
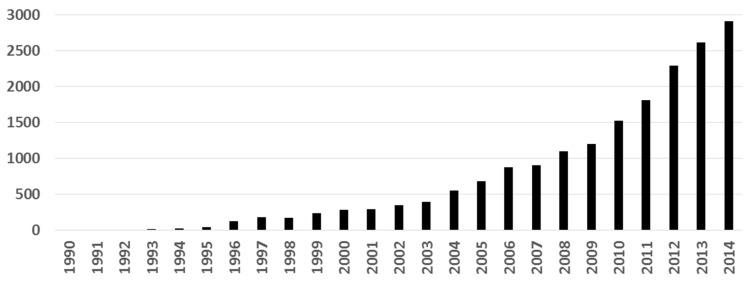
Number of publications where molecular docking was used (search in Scopus).

In the last 25 years, the use of molecular docking has been raised in the context of drug discovery. We searched in Scopus using the word “docking” as a query, along with a selection of the most popular docking softwares according to Kroemer [6] (in the Search field: all fields “docking” and all fields “AutoDock” or “FlexX” or “DOCK” or “FRED” or “Glide” or “GOLD” or “Hammerhead” or “ICM” or “LigandFit” or “QXP” or “SLIDE” or “Surflex”). Figure 1 shows the results of this inquiry: research papers where molecular docking has been used have increased almost exponentially. Interestingly, the number of papers using docking has doubled from 2010 to 2014. This behavior reflects the growing interest of docking methods for structural biology, medicinal chemistry, drug design, and other areas.

Particularly, the challenges of docking are: (i) the prediction of ligands proper orientation; (ii) the prediction of the binding energies; and (iii) the prediction of novel, effective drugs by using the structural knowledge obtained from the models. Nowadays, docking is recognized as the most important theoretical method to determine the orientation of the ligands inside a binding site. Docking protocol explores several poses corresponding to multiple ligand conformations and orientations within the target binding site, and typically detects the right orientations or poses among these orientations [7]. However, the biggest challenge of the docking method (and also its principal problem) is the accurate prediction of binding energies, which has major implications for the prediction of novel effective drugs. This process is performed by using scoring functions that score the predicted poses [8].

Several classes of scoring functions [9] which consider the protein as a rigid body [10,11], or as a soft body [12,13,14] have been developed. They also may consider flexible side chains [13,15] or certain flexible domains in the target [16,17,18]. In general, scoring functions try to reproduce experimental binding affinities, but the ones that come with popular docking programs do not always yield the best predictions. An effort to improve affinity prediction is typically performed using a rescoring process with other simple functions or solvated-based scoring functions. The poses generated by the docking program are taken, and methods such as MM/PBSA (Molecular Mechanics/Poisson-Boltzmann Surface Area) and MM/GBSA (Molecular Mechanics/Generalized-Born Surface Area) [19,20,21], which include an implicit solvent, can be used in order to correct scoring function values and improve docking accuracy. Another strategy is the use of molecular dynamics (MD) simulation to get conformational sampling of the complex obtained by using docking, and subsequent calculation of the binding energy by averaging the score values for different poses extracted from the trajectory [22,23]. Under this approach, the receptor flexibility and the presence of water molecules contribute to a more realistic description of the complex, which could have an influence in binding energy calculations.

It is clearly accepted that scoring functions, which come with popular docking programs, are not good predictors (when rescoring process is not performed). In other words, common molecular docking programs should not be used for accurately predicting binding affinity energy values. However, it is common to find in literature their use for comparing two, or a small number of, complexes. In many reports, the trend when comparing two or a small number of theoretical binding energy values using docking, has agreed with the experimental ones, but perhaps it was a chance coincidence. For instance in many articles, common molecular docking studies have revealed a correlation between score and biological activities for enantiomers. Enantiomers are very similar compounds: they contain the same chemical groups, but have different three-dimensional spatial disposition between them. Considering this, an appropriate prediction of their affinities for a protein target by using simple docking methods is questionable. With this in mind, we selected several enantiomeric ligands and tested if a common docking method is able to identify the most active among them.

## 2. Docking Binding Energy Predictions for Enantiomeric Drugs

### 2.1. Docking Binding Energy Predictions for Enantiomeric Drugs in Literature

We searched for reports where the biological activities of enantiomeric drugs were compared by using just classic docking methodologies. Since enzymes and receptors are chiral, they bind one enantiomer better than the other. As a result, different physiological properties may be associated with each enantiomer; thus, enantiomers could have the same biological activities (with equal or different degrees) or very different biological activities.

Despite that, there is broad evidence that common docking methods fail when used for binding energy predictions; there are also many reports which used docking to calculate binding energies for enantiomers that bind to the same target with similar or different biological activity values. In most cases, authors found a correlation with experimental results. Some examples are mentioned below.

In a recent work, Beavers *et al.* [24] found a cathepsin L inhibitor containing a diacyl hydrazine functionality and one stereogenic center. They observed that this compound was most active than the S-enantiomer, with an IC_50_ of 56 nM, while the R-enantiomer displayed only weak activity (33 µM). Authors performed docking of both enantiomers into the binding site of papain using Glide (New York, NY, USA) due to similitude between papain and cathepsin L binding sites. The highest-scoring pose obtained for the S-enantioner had a binding energy of −9.03 kcal/mol. Meanwhile, the R-enantiomer had a docking score of −7.02 kcal/mol, lower than the score for the S-enantioner. In this sense, a correlation between score and biological activity for both thiocarbazate enantiomers was theoretically obtained. In another report, Kaur *et al.* [25] performed molecular docking of the antileishmanial drug monastrol into the active site of a *Leishmania donovani* pteridine reductase (LdPTR1) homology model using QUANTUM 3.3 docking software (AKos GmbH, Steinen-Schlächtenhaus, Germany). The docking results indicated that both enantiomers have almost the same binding affinity, with binding free energy of −24.92 and −24.20 kJ/mol for the best conformers of monastrol (R) and (S) enantiomers, respectively. Monastrol enantiomers were also docked into the active site of human Eg5/kinesin. Monastrol (R) and (S) enantiomers showed binding free energy of −14.35 and −12.76 kJ/mol for the best conformer, respectively. These values, which were comparable to the IC_50_ values for LdPTR1, displayed greater affinity of monastrol for LdPTR1. These comparisons were used to confirm that PTR1 is the target for the antileishmanial property of monastrol (authors also obtained confirmatory evidence by the PTR1 recombinant enzyme inhibition assay). In other work, Grulich *et al.* [26] constructed the homology model of penicillin G acylase (PGA) from *Achromobacter* sp. and performed molecular docking using AutoDock Vina (La Jolla, CA, USA) to understand molecular basis of PGA enantioselectivity. Authors used enantiomeric forms of seven substrates studied experimentally as ligands. The binding energies predicted by molecular docking strongly correlated (*R* = −0.76) with the molecular weight of investigated substrates. However, the observed difference between binding affinities of individual enantiomers was negligible without any significant influence on the enantioselectivity of PGA. In other report, Han *et al.* [27] evaluated the influence of the stereochemistry on the biological activities of *cis*-cyclopropyl (Cp) abscisic acid (ABA) analogs, 2S,3SCpABA and 2R,3R-CpABA. Authors used molecular docking to confirm the importance of stereochemistry for the interaction of the compounds with the binding site of ABA receptor PYL10, in agreement with the bioassay data. In other report, Malcomsom *et al.* [28] studied the affinities of cyclopropylamine enantiomers against MAO-A and MAO-B using docking (AutoDock, La Jolla, CA, USA). They found a small difference between the (1R,2S) and (1S,2R) enantiomers, and concluded that racemic *cis*-cyclopropylamines can be used for the experimental work based on the lack of enantiomeric differences obtained by using docking. In other work, Chen *et al.* [29] used docking (AutoDock) to study the interactions between mexiletine enantiomers and the D5 variant of monoamine oxidase from *Aspergillus niger* (MAO-N-D5). They found that the calculated binding energies of (R)-mexiletine and (S)-mexiletine with MAO-N-D5 were −5.91 and −6.84 kcal/mol, respectively, which indicates that MAO-N-D5 binds and reacts preferentially with (S)-mexiletine. In other work, Ibrahim *et al.* [30], reported four series of condensed pyrrolo[1,2-*c*]pyrimidines as PI3Kα inhibitors (p110α isoform). They studied the affinity of the reported compounds to the target enzyme by comparing their binding free energy (docking score) and their binding mode with the one of the co-crystallized ligand PI-103. They found that all isomers of the designed compounds displayed comparable docking scores and binding modes similar to that of PI-103. Authors also evaluated the potential effect of compounds’ chirality on inhibitory activity using docking, but results did not reveal significant differences between R and S enantiomers of the studied compounds. In another report, Wang *et al.* [31] designed, synthesized, and evaluated isomers SS, RR, RS, and SR of 3-(4-aminobutyl)-6-(1*H*-indole-3-ylmethyl)piperazine-2,5-dione as urokinase-PA (u-PA) inhibitors. They used docking to compare the designed compounds with known u-PA inhibitors; docking scores gave the SS, RR, RS, and SR higher scores as a result, and approved their interactions with the amino acid residues of the active site fitting the request of u-PA inhibitors. In another work, Eryanni-Levin *et al.* [32] studied the interaction among the 5,6-dihydroxylactone enantiomers in the catalytic region of paraoxonase 1 (PON1). They performed docking calculations and obtained that the binding energies of the interaction for the (S) and (R) enantiomers were −5.57 and −3.88 kcal/mol, respectively, suggesting that there is a great affinity for the S enantiomer.

Summarizing, in all cases presented, docking was used to compare the interaction between enantiomeric forms and a relevant target. Adequate results were obtained in all cases: docking results match with the results observed experimentally. However, we are concerned about the reliability of these results, since it is known that common docking scoring functions are not good predictors of binding energy values.

### 2.2. Testing Docking Binding Energy Predictions for Enantiomeric Drugs

To delve into the aforementioned question, an experiment was elaborated to test the capacity of a popular docking program for predicting binding affinities of enantiomers. One-hundred forty-one enantiomeric pairs with activities against the molecular targets acetylcholinesterase (AChE), butyrylcholinesterase (BChE), monoamine oxidase A (MAO-A), monoamine oxidase B (MAO-B), angiotensin I converting enzyme (ACE), neutral endopeptidase (NEP), and endothelin converting enzyme I (ECE) were selected. The selected pairs were extracted from literature with their biological activities, and are presented in the supplementary material (Table S1) with their respective reported activities [33,34,35,36,37,38,39,40,41,42,43,44,45,46,47,48,49,50,51,52,53,54,55,56,57,58].

Glide and Autodock methods were used for testing. We used the three Glide docking precision options for docking: High-throughput virtual screening (HTVS), standard precision (SP), and extra precision (XP). HTVS is intended for a fast screening of a large number of ligands; this option has a more restricted conformational sampling than SP and XP options. The SP mode is recommended for docking tens to hundreds of thousands of ligands with high accuracy, and the XP mode is recommended for small ligand libraries, where further elimination of false positives is accomplished by a more extensive sampling and advanced scoring [59]. SP is recognized for its “softer” function in order to minimize false negative results, and is the most accurate when identifying ligands inclined to bind, even in cases in which the Glide pose has significant imperfections. XP is a “harder” function, which accurately penalizes poses when they violate established physicochemical principles, such as when charged polar groups are adequately exposed to solvents. In XP mode, the main goal of is to rank (semi-quantitatively) the ligand ability to bind for a specific receptor conformation. The XP scoring function has the following contributions to binding affinity: (a) water displacement by the ligand from “hydrophobic regions” of the receptor binding site; (b) receptor-ligand hydrogen-bonding interactions, as well as other strong electrostatic interactions such as salt bridges; (c) desolvation effects; (d) entropic effects due to the binding restriction of the flexible receptor motion or ligand groups; and (e) metal–ligand interactions. In addition, we used AutoDock Vina [60] as an additional docking method. This method is a popular molecular docking tool available for virtual screening. It is free and many researchers consider that this method ensures high-quality results.

Glide docking modes and Autodock Vina were used to evaluate the accuracy in activity predictions of the enantiomeric pairs against their protein targets. For this purpose, ligand pairs were categorized by subtracting the S enantiomer activity value from the R enantiomer activity value. For each pair of enantiomers we compared the experimental activities A(R) and A(S) (experimental activities of the R and S enantiomers, respectively) to get the value *C*^A^, where *C*^A^ = A(R) − A(S). *C*^A^ adopts three categories: *C*^A^ = *D*^R^ when A(R) − A(S) > 0.5, *C*^A^ = *D*^S^ when A(S) − A(R) > 0.5 and *C*^A^ = *E* when |A(R) − A(S)| ≤ 0.5. We made the same comparisons for the predicted activities P(R) and P(S) (predicted activities of the R and S enantiomers respectively using docking) to get the value *C*^P^, where *C*^P^ = P(R) − P(S). C^P^ also adopts three categories: *C*^P^ = *D*^R^ when P(R) − P(S) > 0.5, *C*^P^ = *D*^S^ when P(S) − P(R) > 0.5, and *C*^P^ = *E* when |P(R) − P(S)| ≤ 0.5. The values *D*^R^, *D*^S^, and *E* were used to decide whether the docking predictions are correct or not. We consider that prediction is correct if *C*^A^ = *C*^P^.

Then, we defined a match if categories of the comparison *E*, *D*^R^ or *D*^S^ correspond to the experimental activities and predictions. Additionally, we defined a mismatch if categories of the comparison *E*, *D*^R^, or *D*^S^ differ for experimental activities and predictions. Finally, we calculated the match and mismatch percent for three replies and determined the associated error.

The 141 enantiomeric pairs include 37 cholinesterase (ChE) inhibitors, 37 monoamine oxidase (MAO) inhibitors, 35 ACE inhibitors, 17 NEP inhibitors, and 15 ECE inhibitors. The percentages of match and mismatch cases for each target and for the full dataset are reported in Table 1. Full dataset results and individual results display a greater mismatch percentage indicating that molecular docking method was not reliable for accurately predicting targets preference for R or S enantiomers. The match and mismatch percentages for most of the cases were comparable to the result of assigning random categories to each enantiomers comparison. If the three categories are randomly assigned to each case, there are one-third of the cases (33.33%) with a right assignment and two-third of the cases (66.67%) with wrong assignments. Match percent was similar or lesser than 33.33% for almost all of the studied targets and for the full dataset. The exception in Table 1 is the match percent of ACE inhibitors obtained using Glide SP; however, a match percent of 44.44% is not too encouraging. The results in Table 1 show that predictions of the differential potency of enantiomeric pairs using Glide and Autodock Vina methods have a random behavior. Therefore, when the method precisely identifies which enantiomer presents higher binding affinity is due to chance.

It would be useful to look in detail an apparently positive result: For instance, ACE inhibitors SRS(R)R and SRS(S)R (supplementary material, Table S1) have IC_50_ = 1.0 × 10^5^ and 54 nM, respectively. Docking score values using XP and SP correlate with the experimental ones: XP: −6.75 kcal·mol^−1^ for SRS(R)R and −8.69 kcal·mol^−1^ for SRS(S)R, SP: −5.69 kcal·mol^−1^ for SRS(R)R and −7.84 kcal·mol^−1^ for SRS(S)R. In contrast, HTVS mode predicts both enantiomers as equals. Considering the analysis exposed here, the positive predictions obtained using XP and SP modes are not reliable.

## 3. Docking Binding Energy Predictions for Pairs of Drugs with Different Molecular Weight

We observed that docking was not able to correctly predict the differential binding energies of enantiomers, which are molecular systems that have the same molecular weight (*M*_W_). We consider relevant to test if docking gives some advantage to drugs with a higher or lower *M*_W_. For this, we performed the same study using 202 pairs with small differences in *M*_W_ due to lengthening of aliphatic chains and/or different chemical functions. This dataset includes compounds with biological activities against the same molecular targets previously studied. The selected pairs and their biological activities are presented in the supplementary material (Table S2) [33,35,36,44,45,61,62,63,64,65,66]. HTVS, SP, and XP Glide docking modes, and Autodock Vina, were used to evaluate the accuracy in prediction of biological activities of *M*_W_ pairs against the selected protein targets.

Ligand pairs were categorized by subtracting the higher *M*_W_ ligand activity value from the lower *M*_W_ ligand activity value. For each pair of ligands we compared the experimental activities A(H) and A(L) (experimental activities of the higher and lower *M*_W_ ligands, respectively) to get the value *C*^A^, where *C*^A^ = A(H) − A(L). *C*^A^ adopts three categories: *C*^A^ = *D*^H^ when A(H) − A(L) > 0.5, *C*^A^ = *D^L^* when A(L) − A(H) > 0.5 and *C*^A^ = *E* when |A(H) − A(L)| ≤ 0.5. We made the same comparisons for the predicted activities P(H) and P(L) (predicted activities of the higher and lower *M*_W_ ligands, respectively, using docking), to get the value *C*^P^, where *C*^P^ = P(H) − P(L). C^P^ also adopts three categories: *C*^P^ = *D*^H^ when P(H) − P(L) > 0.5, *C*^P^ = *D*^L^ when P(L) − P(H) > 0.5, and *C*^P^ = *E* when |P(H) − P(L)| ≤ 0.5. The values *D*^H^, *D*^L^, and *E* were used to decide whether the docking predictions are correct or not. We consider that prediction is correct if *C*^A^ = *C*^P^.

Then, we defined a match if categories of the comparison *E*, *D*^H^, or *D*^L^ coincide for experimental activities and predictions. Furthermore, we defined a mismatch if categories of the comparison *E*, *D*^H^ or *D*^L^ differ for experimental activities and predictions. Finally, we calculated the percent of match and mismatch for three replies, and determined the associated error.

The 202 pairs with different *M*_W_ include 86 ChE inhibitors, 39 MAO inhibitors, 28 ACE inhibitors, 28 NEP inhibitors, and 21 ECE inhibitors. Percentages of match and mismatch cases for each target and for the full dataset are reported in Table 2. Full dataset results and individual results show a greater mismatch percentage indicating that molecular docking method was not reliable for correctly predicting targets’ preferences for the most active ligand. The match and mismatch percentages for most of the cases were comparable to the result of assigning random categories to each ligand comparison. As in the comparison of enantiomer pairs, match percent was similar or lesser than 33.33% for almost all of the studied targets and for the full data set. The exceptions in Table 2 are the match percent of ACE inhibitors obtained using Glide HTVS, match percent of NEP inhibitors obtained using Glide XP, and match percent of MAO inhibitors obtained using Glide XP; however, match percent values of 42.86%, 41.07%, and 44.87% are not too encouraging. A special case was observed for NEP inhibitors using Glide SP that has a match percent value of 63.10%. Overall, results in Table 2 show that predictions for the differential potency of the studied pairs by using Glide methods have a random behavior. Therefore, as in the analysis of enantiomer pairs, when the method accurately identifies which ligand presents higher binding affinity is due to chance.

In addition, we tested if docking has a preference in energy for higher or lower *M*_W_ ligands. The results of this analysis are reported in Table 3. The lower *M*_W_ ligand has the best experimental activity for the 65.35% of the 202 pairs. However, docking predicts that over 50% of lower *M*_W_ ligands have the best activity. Once again, the behavior of this test seems to be random, and docking did not predict the correct trend, but it does not have a preference in energy for higher or lower *M*_W_ ligands.

## 4. Discussion and Recommendations

In the last years, an increasing number of experimentalists have used docking calculations for supporting their research results. Increased deposition of protein-ligand X-ray crystal structures is one factor that supports the expansion for the usage of this method. It is easy to demonstrate the utility of docking due to its ability to reproduce experimentally-known ligand poses in a reliable manner. At the same time, rational analysis of interactions between active poses and the protein target is an initial point for drug design. However, regarding affinity prediction, some important aspects should be considered.

Docking methods can be used to identify potential molecules with high affinities after exploring a broad dataset. The quality of scoring function is very important during this process; the most common docking programs have optimal scoring functions for these tasks. At the identification stage (hits searching), it is possible to find molecules with a weak activity that could be used as a source to initiate a medicinal chemistry lead optimization phase. During the identification stage docking is very useful, but it is not common to get good results in the lead optimization phase using docking.

It is not common to find nanomolar compounds when the identification stage is performed; therefore, compounds found during this stage should be chemically transformed to get a lead compound. The use of docking programs for the leading optimization stage, e.g., to transform the identified scaffold to get more potent compounds, requires a scoring function able to distinguish potent ligands from moderately active and inactive ones. However, common docking scoring functions do not discriminate correctly between true potent and weak active compounds. A successful discrimination could be improved by rescoring of the best configuration identified during docking.

Comparing the binding energies of two ligands for a target using popular docking methods is not an uncommon practice. However, our results based on this kind of comparison by using different pairs, different targets, and two different programs, show that predictions do not match with the experimental data. This is not unexpected; several benchmarking studies indicate that docking scoring functions poorly predict the affinities of ligands for their targets [67,68,69,70]. Scoring functions are able—at a coarse level—to classify compounds into actives and inactives; but they are not reliable for pose-ranking and fail in potency prediction. The purpose of docking scoring is a rank ordering of the ligands based on their docked scores, such that the scores correlate with experimental binding affinities. Under this context, the prediction of the pair of ligands affinities considers solely the ranking of two points of the correlation, but two points do not allow the assessment of the quality of the ranking. Therefore, correct predictions of more cases should be necessary to guarantee a successful rank ordering.

In general, enantiomers have similar biological activities or have different not detected activities by using the simple scoring functions contained in common docking programs (usually, one enantiomer is very active and the other is weakly active). Therefore, it is not possible, in general, to identify between the two enantiomers, which is the most active one using docking. Our test demonstrated that correct predictions are not reliable when pairs are compared. A suggestion arises from the observations mentioned above. When enantiomer pairs are compared using docking, the assessment of the protein–ligand predictions for the system under study is recommended. It is not enough to calculate the binding energies for enantiomers; in addition, calculations of the binding energies for other similar ligands should be performed. If the predictions are good for the enantiomers and a set of congeneric ligands (a good correlation), then, one might argue that the prediction of the comparison between the studied enantiomers is reliable.

## 5. Materials and Methods

### 5.1. Datasets and Molecular Structures

Two datasets were generated. The first one includes 141 enantiomeric pairs with activities against some selected molecular targets. The second one includes 202 pairs with small differences in *M*_W_ with activities against the same molecular targets. The selected compounds have biological activities against the molecular targets AChE, BChE, MAO-A, MAO-B, ACE, NEP, and ECE [33,34,35,36,37,38,39,40,41,42,43,44,45,46,47,48,49,50,51,52,53,54,55,56,57,58,61,62,63,64,65,66]. All the studied ligands in this work were sketched using Maestro Suite and prepared using LigPrep with the force field OPLS_2005 [71]. The biological activities were converted to logarithmic scale for comparison with calculated binding energies.

The structural files of the following protein targets were downloaded from the RCSB Protein Data Bank repository: *human* AChE (hAChE) PDB ID: 4M0E [72]; *mus* musculus AChE (mAChE) PDB ID: 2HA2 [73]; *human* BChE (hBChE) PDB ID: 1POI [74]; *human* MAO-A (hMAO-A) PDB ID: 2BXR [75]; *rattus norvegicus* MAO-A (mMAO-A) PDB ID: 1O5W [76]; *human* MAO-B (hMAO-B) PDB ID: 1S3E [77]; *human* ACE (hACE) PDB ID: 1O86 [78]; *human* NEP (hNEP) PDB ID: 2QPJ [79]; and *human* ECE (hECE) PDB ID: 3DWB [80]. All targets were processed with the Protein Preparation Wizard in the Schrödinger Suite [81]. Hydrogen atoms were added followed by the adjustment of bond orders. The protonation and tautomeric states for protonable residues were adjusted to match pH = 7.4. Missing residues and loop segments near the active site were added by using Prime [82]. Water molecules beyond 5.0 Å from the active site were deleted. Proteins were finally subjected to geometry optimization by using OPLS_2005 force field [71].

### 5.2. Docking Methodology

Docking tests were performed using the software Glide [12] and Autodock Vina [60]. Glide offers a complete solution for ligand–receptor docking and is widely used for drug discovery [83,84], virtual screening [85,86], structure-activity relationship analysis [87,88,89], pharmacophore modeling [90,91,92], evaluation of enzymatic reaction pathways [93,94], and other studies. All grid boxes for molecular docking were centered in the ligand position coming from the crystal structures. The grid boxes’ dimensions were 35 × 35 × 35 Å in order to include all binding sites. High-throughput virtual screening (HTVS), standard precision (SP), and extra precision (XP) Glide modes were proved.

Default docking parameters were used. Glide docking uses hierarchical filters to find the best ligand binding locations in the defined receptor grid space. The filters include positional, conformational, and orientational sampling of the ligand and subsequent energy evaluation of the interactions between the ligand and the protein. Ligand minimization in the receptor field is carried out using the OPLS-AA force field [71] with a distance-dependent dielectric of 2.0. Afterward, the lowest energy poses are subjected to a Monte Carlo (MC) procedure that samples the nearby torsional minima. The best pose for a given ligand is determined by the GlideScore score [95], including terms for buried polar groups and steric clashes.

Autodock Vina parameters were defined in a similar way as in Glide. Grid boxes dimensions were 35 × 35 × 35 Å. Autodock Vina implements an efficient scoring function optimization algorithm for estimating protein-ligand affinity and a search algorithm for predicting the plausible binding modes [60]. Vina repeats the calculations several times with different randomizations, it can be performed in parallel with a multicore machine.

## 6. Conclusions

Glide and AutoDock Vina docking energies for two complexes between a target protein and enantiomer pairs were determined and compared. The capacity to reproduce the trend observed in experimental affinity values were tested for 141 enantiomeric pairs, including compounds with biological activities reported against several protein targets. We found that predictions failed for all targets. We concluded that common molecular docking methods cannot perform successful predictions for enantiomer pairs. We found the same result for pairs of compounds with different molecular weights. The purpose of this article is to alert the readers about the unreliability of binding energy comparisons between pairs of molecules using docking. 

## Figures and Tables

**Table 1:** Percentages of match and mismatch for the categories *E*, *D*^R^, or *D*^S^ in the comparison between *C*^A^ and *C*^P^ for enantiomer pairs.

Target	Results	Glide HTVS	Glide SP	Glide XP	Autodock Vina
ChE	Match %	28.70	8.11	24.32	2.78
	Mismatch %	71.30	91.89	75.68	97.22
	Error	3.21	4.68	3.82	0.00
	Total pairs	36	36	37	36
MAO	Match %	22.86	18.92	31.08	5.41
	Mismatch %	77.14	81.08	68.92	94.59
	Error	2.86	2.70	1.91	0.00
	Total pairs	35	37	37	37
ACE	Match %	25.29	37.14	28.57	24.76
	Mismatch %	74.71	62.86	71.43	75.24
	Error	3.98	5.71	8.08	6.60
	Total pairs	29	35	35	35
NEP	Match %	21.57	7.84	20.59	31.37
	Mismatch %	78.43	92.16	79.41	68.63
	Error	6.79	3.40	12.48	13.58
	Total pairs	17	17	17	17
ECE	Match %	26.19	44.44	23.33	15.56
	Mismatch %	73.81	55.56	76.67	84.44
	Error	4.12	10.18	14.14	7.70
	Total pairs	14	15	15	15
Total	Match %	25.19	22.14	25.53	13.81
	Mismatch %	74.81	77.86	74.47	86.19
	Error	0.00	3.78	3.75	2.18
	Total pairs	131	140	141	140

HTVS: High-throughput virtual screening, SP: standard precision, XP: extra precision, ChE: cholinesterase, MAO: monoamine oxidase, monoamine oxidase, ACE: angiotensin I converting enzyme, NEP: neutral endopeptidase, ECE: endothelin converting enzyme I.

**Table 2:** Percentages of match and mismatch for the categories *E*, *D*^H^, or *D*^L^, when comparing C^A^ and C^P^ for *M*_W_ pairs.

Target	Results	Glide HTVS	Glide SP	Glide XP	Autodock Vina
ChE	Match %	34.65	18.57	30.25	7.82
	Mismatch %	65.35	81.43	69.75	92.18
	Error	4.62	1.93	14.84	3.97
	Total pairs	76	79	81	81
MAO	Match %	20.18	31.58	44.87	40.35
	Mismatch %	79.82	68.42	55.13	59.65
	Error	6.08	2.63	12.69	1.52
	Total pairs	38	38	39	39
ACE	Match %	42.86	25.00	14.29	16.67
	Mismatch %	57.14	75.00	85.71	83.33
	Error	8.25	0.00	0.00	10.31
	Total pairs	21	28	28	28
NEP	Match %	33.33	63.10	41.07	20.24
	Mismatch %	66.67	36.90	58.93	79.76
	Error	15.28	4.12	2.53	2.06
	Total pairs	10	28	28	28
ECE	Match %	25.40	33.33	35.71	19.05
	Mismatch %	74.60	66.67	64.29	80.95
	Error	11.98	12.60	3.37	4.76
	Total pairs	21	21	21	21
Total	Match %	31.33	30.07	32.15	18.37
	Mismatch %	68.67	69.93	67.85	81.63
	Error	3.95	1.81	6.76	1.84
	Total pairs	166	194	197	196

**Table 3:** Predictions of higher and lower *M*_W_ ligand pairs.

Set of Ligands	Experimental	Glide HTVS	Glide SP	Glide XP	Autodock Vina
Higher *M*_W_ %	34.65	53.21	52.75	51.27	57.82
Lower *M*_W_ %	65.35	46.79	47.25	48.73	42.18
Error	-	3.68	2.84	4.65	2.30
Total pairs	202	166	194	197	196

## Supplementary Materials

Supplementary materials can be found at http://www.mdpi.com/1422-0067/17/4/525/s1.

## Abbreviations

AChE	acetylcholinesterase
BChE	butyrylcholinesterase
MAO	monoamine oxidase
ACE	angiotensin I converting enzyme
NEP	neutral endopeptidase
ECE	endothelin converting enzyme I
