# Supplementary material for: Is It Reliable to Use Common Molecular Docking Methods for Comparing the Binding Affinities of Enantiomer Pairs for Their Protein Target?
Source: Int J Mol Sci. 2016 Apr 20;17(4):525. doi: 10.3390/ijms17040525 (PMC4848981; doi:10.3390/ijms17040525)
Supplement: Supplementary file 1 [file ijms-17-00525-s001.pdf]

Supplementary Materials: Is It Reliable to Use Common Molecular Docking Methods for Comparing the Binding Affinities of Enantiomer Pairs for Their Protein Target?

David Ramírez and Julio Caballero

Table S1. Ligand dataset collection of enantiomers.

Target	Compound ID	Activity		Reference
		R/(+)	S/(−)	
hAChE	VX	1.180×10^{-2} [a]	1.370 [a]	[52]
	nc-VX	2.000×10^{-6} [a]	1.100×10^{-4} [a]	[52]
	Bambuterol	4.300×10^{2} [a]	1.600×10^{-1} [a]	[37]
	a(EMP–CHMC)	1.200×10^{6} [a]	2.800×10^{7} [a]	[33]
	b(nPrMP–CHMC)	6.100×10^{6} [a]	1.580×10^{8} [a]	[33]
	1	2.000×10^{2} [b]	1.000×10^{4} [b]	[48]
	2	2.600×10^{1} [b]	8.000×10^{1} [b]	[48]
	3	1.422×10^{2} [b]	1.020×10^{2} [b]	[48]
	HupA	3.153×10^{3} [c]	6.500×10^{1} [c]	[58]
	18a	1.400×10^{1} [c]	6.090×10^{2} [c]	[55]
	Malaoxon	1.270×10^{5} [a]	1.090×10^{4} [a]	[53]
	Dizocilpine	2.000×10^{-1} [b]	6.200×10^{-3} [b]	[42]
	Cycloheptyl–CH_3	7.600×10^{2} [a]	2.200×10^{5} [a]	[35]
	Cycloheptyl–C_2H_5	1.400×10^{1} [a]	6.500×10^{4} [a]	[35]
	Cycloheptyl–C_4H_9	3.200×10^{1} [a]	9.400×10^{4} [a]	[35]
	Cycloheptyl–C_5H_{11}	1.290×10^{2} [a]	3.400×10^{5} [a]	[35]
	Cycloheptyl–C_6H_{13}	1.140×10^{2} [a]	2.900×10^{5} [a]	[35]
	Isopropyl–CH_3	1.500×10^{1} [a]	3.170×10^{2} [a]	[35]
	Isopropyl–C_2H_5	2.900×10^{1} [a]	7.500×10^{1} [a]	[35]
	Isopropyl–C_5H_{11}	2.600×10^{3} [a]	1.500×10^{3} [a]	[35]
	Isopropyl–C_6H_{13}	3.000×10^{1} [a]	3.600×10^{3} [a]	[35]
	Cycloheptyl	1.400×10^{6} [a]	3.000×10^{8} [a]	[35]
	Isopropyl	8.700×10^{4} [a]	1.300×107 [a]	[35]
	3,3-Dimethylbutyl	3.300×10^{7} [a]	1.000×109 [a]	[35]
	Limonene	2.200×10^{-1} [d]	2.500×10^{-1} [d]	[51]
	Terpinen-4-ol	2.440×10^{-1} [d]	2.140×10^{-1} [d]	[51]
	Carvone	1.850×10^{6} [c]	1.380×10^{6} [c]	[51]
	Profenofos	1.000×10^{3} [c]	7.180×10^{4} [c]	[43]
	Crotoxyphos	1.900×10^{3} [c]	1.000×10^{3} [c]	[43]
mAChE	Bambuterol	7.900×10^{2} [a]	7.700×10^{-1} [a]	[37]
	P-CHMPTCh	8.100×10^{-1} [a]	1.900×10^{2} [a]	[47]
	iPrMPTCh	1.400×10^{-1} [a]	1.600×10^{1} [a]	[47]
	DMBMPTCh	1.900×10^{1} [a]	3.600×10^{2} [a]	[47]
hBChE	Bambuterol	6.400×10^{6} [a]	1.200×10^{6} [a]	[37]
	HupA	2.829×10^{5} [c]	5.363×10^{4} [c]	[58]
	Malaoxon	1.460×10^{4} [a]	4.240×10^{3} [a]	[53]
	Dizocilpine	4.500×10^{-1} [b]	1.790×10^{-2} [b]	[42]

Table S1. *Cont.*

Target	Compound ID	Activity		Reference
		R/(+)	S/(−)	
hMAO-A	PwTx-I	3.900×10^{4} c	3.400×10^{4} c	[54]
	11a–11b	3.900×10^{1} c	2.400×10^{1} c	[56]
	11c–11d	1.370×10^{3} c	1.540×10^{3} c	[56]
	11g–11h	3.700×10^{1} c	2.500×10^{1} c	[56]
	11i–11j	6.500×10^{2} c	7.000×10^{1} c	[56]
	3a–3b	1.330×10^{2} b	5.840×10^{2} b	[36]
	4a–4b	1.690×10^{2} b	6.140×10^{2} b	[36]
	5a–5b	1.130×10^{1} b	1.816×10^{2} b	[36]
	FLA 336	9.000×10^{-1} b	4.000×10^{-1} b	[39]
	7–8	4.800 c	6.800 c	[40]
	3	3.100×10^{1} b	2.840×10^{2} b	[34]
	7	7.700×10^{1} b	1.600×10^{2} b	[34]
	9	6.000 b	1.860×10^{2} b	[34]
	11	2.000 b	1.020×10^{2} b	[34]
	1	1.300×10^{-2} b	5.000×10^{-2} b	[38]
	4	1.200×10^{-2} b	5.000×10^{-3} b	[38]
	SAL	3.610×10^{1} b	8.130×10^{1} b	[50]
	NM-SAL	3.790×10^{1} b	1.495×10^{2} b	[50]
	5–6	7.300×10^{-1} b	2.600 b	[57]
	7–8	1.800 b	1.100 b	[57]
	11–12	1.300 b	6.900×10^{-1} b	[57]
	13–14	1.800×10^{-1} b	7.200×10^{-1} b	[57]
mMAO-A	Amphetamine	2.030×10^{2} b	3.380×10^{1} b	[39]
	Methamphetamine	9.900×10^{1} b	1.100×10^{2} b	[39]
	Deprenyl	7.600×10^{1} b	1.400×10^{1} b	[39]
hMAO-B	PwTx-I	1.500×10^{4} c	8.000×10^{3} c	[54]
	11a–11b	1.229×10^{4} c	1.681×10^{4} c	[56]
	11c–11d	2.900×10^{4} c	8.470×10^{4} c	[56]
	11g–11h	1.917×10^{4} c	2.120×10^{4} c	[56]
	11i–11j	3.400×10^{4} c	8.007×10^{4} c	[56]
	3a–3b	1.410×10^{1} b	1.560×10^{2} b	[36]
	4a–4b	4.780×10^{1} b	1.490×10^{2} b	[36]
	5a–5b	2.490×10^{1} b	4.490×10^{1} b	[36]
	FLA 336	2.500×10^{1} b	2.100×10^{-2} b	[39]
	7–8	1.000×10^{1} b	2.700 b	[40]
	1	2.700×10^{-3} b	1.000×10^{-3} b	[38]
	4	1.400 b	2.400 b	[38]

Table S1. *Cont.*

Target	Compound ID	Activity		Reference
		R/(+)	S/(−)	
	SS(R)SS–SS(S)SS	1.100 [c]	1.500 [c]	[49]
	S(R)SSS–S(S)SSS	1.200 [c]	1.500 [c]	[49]
	SRRS(R)–SRRS(S)	3.980×10^{1} [c]	3.300 [c]	[49]
	SSSS(R)–SSSS(S)	1.220×10^{1} [c]	1.500 [c]	[49]
	S(R)RSR–S(S)RSR	3.980×10^{1} [c]	2.940×10^{1} [c]	[49]
	SRS(R)R–SRS(S)R	1.000×10^{5} [c]	5.400×10^{1} [c]	[49]
	RR(R)SS–RR(S)SS	1.000×10^{5} [c]	1.080×10^{2} [c]	[49]
	SSS(R)S–SSS(S)S	1.100×10^{3} [c]	1.500 [c]	[49]
	(R)SSSS–(S)SSSS	1.900×10^{3} [c]	1.500 [c]	[49]
	SS(R)RR–SS(S)RR	2.600×10^{3} [c]	1.000×10^{5} [c]	[49]
	RRS(R)R–RRS(S)R	1.000×10^{5} [c]	5.500×10^{3} [c]	[49]
	S(R)RRS–S(S)RRS	3.980×10^{1} [c]	7.100×10^{3} [c]	[49]
	RR(R)RS–RR(S)RS	1.000×10^{5} [c]	7.800×10^{3} [c]	[49]
	R(R)RRR–R(S)RRR	1.000×10^{5} [c]	2.300×10^{4} [c]	[49]
	(R)RRRR–(S)RRRR	1.000×10^{5} [c]	3.300×10^{4} [c]	[49]
	(R)SSSR–(S)SSSR	3.600×10^{4} [c]	1.220×10^{1} [c]	[49]
	RSR(R)R–RSR(S)R	2.300×10^{4} [c]	4.700×10^{4} [c]	[49]
**hACE**	RSR(R)S–RSR(S)S	1.000×10^{5} [c]	6.000×10^{4} [c]	[49]
	4a(2R,3R)–4b(2S,3S)	8.000×10^{-2} [b]	1.600×10^{-2} [b]	[41]
	4c(2S,3R)–4d(2R,3S)	4.200×10^{-3} [b]	9.500×10^{-2} [b]	[41]
	8b(2R,3R)–8a(2S,3S)	2.400×10^{-2} [b]	7.000×10^{-2} [b]	[45]
	8c(2S,3R)–8d(2R,3S)	4.600×10^{-3} [b]	3.000×10^{-2} [b]	[45]
	15b(2R,3R)–15a(2S,3S)	5.500×10^{-2} [b]	5.800×10^{-2} [b]	[45]
	15c(2S,3R)–15d(2R,3S)	4.100×10^{-3} [b]	6.600×10^{-2} [b]	[45]
	9b(2R3R)–9a(2S3S)	3.300×10^{-2} [b]	2.900×10^{-2} [b]	[44]
	9c(2S3R)–9d(2R3S)	1.500×10^{-2} [b]	2.900×10^{-1} [b]	[44]
	10a(2S3S)–10b(2R3R)	6.500×10^{-2} [b]	1.300×10^{-1} [b]	[44]
	10c(2S3R)–10d(2R3S)	3.800×10^{-2} [b]	3.300×10^{-1} [b]	[44]
	13b(2R3R)–13a(2S3S)	4.300×10^{-2} [b]	4.100×10^{-2} [b]	[44]
	13c(2S3R)–13d(2R3S)	1.000×10^{-2} [b]	1.700×10^{-1} [b]	[44]
	14b(2R3R)–14a(2S3S)	7.600×10^{-2} [b]	2.600×10^{-2} [b]	[44]
	14c(2S3R)–14d(2R3S)	1.000×10^{-2} [b]	1.340×10^{-1} [b]	[44]
	5f1–5f2	1.400×10^{-3} [b]	3.600×10^{-2} [b]	[46]
	7f1–7f2	4.000×10^{-4} [b]	3.800×10^{-3} [b]	[46]
	8f1–8f2	4.100×10^{-4} [b]	6.500×10^{-4} [b]	[46]

Table S1. *Cont.*

Target	Compound ID	Activity		Reference
		R/(+)	S/(−)	
hNEP	4a(2R,3R)–4b(2S,3S)	2.300×10^{-3} [b]	2.100×10^{-3} [b]	[41]
	4c(2S,3R)–4d(2R,3S)	1.700×10^{-3} [b]	7.000×10^{-4} [b]	[41]
	8b(2R,3R)–8a(2S,3S)	1.300×10^{-3} [b]	3.000×10^{-2} [b]	[45]
	8c(2S,3R)–8d(2R,3S)	2.700×10^{-3} [b]	1.500×10^{-2} [b]	[45]
	15b(2R,3R)–15a(2S,3S)	1.800×10^{-3} [b]	9.400×10^{-3} [b]	[45]
	15c(2S,3R)–15d(2R,3S)	3.800×10^{-3} [b]	1.300×10^{-2} [b]	[45]
	9b(2R3R)–9a(2S3S)	2.400×10^{-3} [b]	2.200×10^{-3} [b]	[44]
	9c(2S3R)–9d(2R3S)	2.500×10^{-3} [b]	5.000×10^{-3} [b]	[44]
	10a(2S3S)–10b(2R3R)	3.000×10^{-2} [b]	1.500×10^{-3} [b]	[44]
	10c(2S3R)–10d(2R3S)	3.500×10^{-4} [b]	5.000×10^{-3} [b]	[44]
	13b(2R3R)–13a(2S3S)	7.000×10^{-4} [b]	1.000×10^{-2} [b]	[44]
	13c(2S3R)–13d(2R3S)	2.100×10^{-3} [b]	2.700×10^{-2} [b]	[44]
	14b(2R3R)–14a(2S3S)	3.000×10^{-3} [b]	8.000×10^{-3} [b]	[44]
	14c(2S3R)–14d(2R3S)	3.000×10^{-3} [b]	4.300×10^{-2} [b]	[44]
	5f1–5f2	1.300×10^{-1} [b]	1.850 [b]	[46]
	7f1–7f2	6.800×10^{-2} [b]	1.000×10^{2} [b]	[46]
	8f1–8f2	1.720×10^{-1} [b]	6.700 [b]	[46]
hECE	8b(2R,3R)–8a(2S,3S)	1.000×10^{-2} [b]	1.460 [b]	[45]
	8c(2S,3R)–8d(2R,3S)	1.000×10^{-1} [b]	1.800×10^{-1} [b]	[45]
	15b(2R,3R)–15a(2S,3S)	1.800×10^{-2} [b]	1.100×10^{-1} [b]	[45]
	15c(2S,3R)–15d(2R,3S)	2.800×10^{-2} [b]	9.000×10^{-2} [b]	[45]
	9b(2R3R)–9a(2S3S)	2.000×10^{-1} [b]	1.000×10^{-1} [b]	[44]
	9c(2S3R)–9d(2R3S)	7.800×10^{-1} [b]	5.800×10^{-1} [b]	[44]
	10a(2S3S)–10b(2R3R)	1.100×10^{-1} [b]	1.350×10^{-1} [b]	[44]
	10c(2S3R)–10d(2R3S)	9.200×10^{-1} [b]	1.900×10^{-1} [b]	[44]
	13b(2R3R)–13a(2S3S)	2.600×10^{-2} [b]	1.140 [b]	[44]
	13c(2S3R)–13d(2R3S)	2.900×10^{-1} [b]	$6.800\ 10^{-1}$ [b]	[44]
	14b(2R3R)–14a(2S3S)	2.500×10^{-1} [b]	$6.700\ 10^{-1}$ [b]	[44]
	14c(2S3R)–14d(2R3S)	1.900×10^{-1} [b]	1.240 [b]	[44]
	5f1–5f2	2.600×10^{-2} [b]	8.000×10^{-3} [b]	[46]
	7f1–7f2	2.400×10^{-1} [b]	7.700×10^{-3} [b]	[46]
	8f1–8f2	2.750×10^{-1} [b]	1.400×10^{-2} [b]	[46]

[a] k_i ($M^{-1} \cdot min^{-1}$); [b] K_i (μM); [c] IC_{50} (nM); [d] % inhibitory activity (1.2 mM).

Table S2. Ligand dataset collection of ligands with different molecular weights.

Target	Compound	Activity		Reference
		Lower M_W	Higher M_W	
hAChE	(S)-EMP–CHMC–(S)-nPrMP–CHMC	2.800×10^7 [a]	1.580×10^8 [a]	[33]
	(R)-EMP–CHMC–(R)-nPrMP–CHMC	1.200×10^6 [a]	6.100×10^6 [a]	[33]
	(S)-Cycloheptyl-CH_3–(S)-Cycloheptyl-C_2H_5	2.200×10^5 [a]	6.500×10^4 [a]	[35]
	(S)-Cycloheptyl-CH_3–(S)-Cycloheptyl-C_4H_9	2.200×10^5 [a]	9.400×10^4 [a]	[35]
	(S)-Cycloheptyl-CH_3–(S)-Cycloheptyl-C_5H_{11}	2.200×10^5 [a]	3.400×10^5 [a]	[35]
	(S)-Cycloheptyl-CH_3–(S)-Cycloheptyl-C_6H_{13}	2.200×10^5 [a]	2.900×10^5 [a]	[35]
	(R)-Cycloheptyl-CH_3–(R)-Cycloheptyl-C_2H_5	7.600×10^2 [a]	1.400×10^1 [a]	[35]
	(R)-Cycloheptyl-CH_3–(R)-Cycloheptyl-C_4H_9	7.600×10^2 [a]	3.200×10^1 [a]	[35]
	(R)-Cycloheptyl-CH_3–(R)-Cycloheptyl-C_5H_{11}	7.600×10^2 [a]	1.290×10^2 [a]	[35]
	(R)-Cycloheptyl-CH_3–(R)-Cycloheptyl-C_6H_{13}	7.600×10^2 [a]	1.140×10^2 [a]	[35]
	2a–2i	5.020×10^1 [c]	6.400×10^2 [c]	[63]
	2b–2g	3.730×10^1 [c]	2.090×10^2 [c]	[63]
	2b–2l	3.730×10^1 [c]	4.150×10^2 [c]	[63]
	2c–2k	4.210×10^1 [c]	2.770×10^2 [c]	[63]
	2c–2m	4.210×10^1 [c]	4.810×10^2 [c]	[63]
	3a–3e	3.640×10^2 [c]	1.730×10^1 [c]	[63]
	3a–3k	3.640×10^2 [c]	5.290×10^2 [c]	[63]
	3b–3f	2.400×10^2 [c]	4.160 [c]	[63]
	3b–3l	2.400×10^2 [c]	6.200×10^1 [c]	[63]
	3c–3g	2.800×10^2 [c]	7.800×10^{-1} [c]	[63]
	3c–3m	2.800×10^2 [c]	4.060×10^1 [c]	[63]
	3d–3h	3.160×10^1 [c]	1.850 [c]	[63]
	3d–3n	3.160×10^1 [c]	3.760×10^1 [c]	[63]
	3a–3e	1.100×10^3 [c]	2.280×10^3 [c]	[64]
	3b–3f	8.200×10^2 [c]	1.360×10^3 [c]	[64]
	3c–3g	2.100×10^2 [c]	1.000×10^2 [c]	[64]
	3d–3h	1.500×10^2 [c]	2.200×10^2 [c]	[64]
	3i–3m	2.660×10^3 [c]	3.180×10^3 [c]	[64]
	3j–3n	1.960×10^3 [c]	3.580×10^3 [c]	[64]
	3k–3o	5.000×10^1 [c]	1.500×10^2 [c]	[64]
	3l–3p	1.400×10^3 [c]	1.300×10^2 [c]	[64]
	1a–1c	2.140×10^3 [c]	4.900×10^2 [c]	[65]
	1a–1f	2.140×10^3 [c]	4.400×10^2 [c]	[65]
	1b–1d	4.200×10^2 [c]	2.700×10^2 [c]	[65]
	1b–1g	4.200×10^2 [c]	3.500×10^1 [c]	[65]
	1c–1f	4.900×10^2 [c]	4.400×10^2 [c]	[65]
	1d–1g	2.700×10^2 [c]	3.500×10^1 [c]	[65]
	1j–1n	2.400×10^2 [c]	1.600×10^2 [c]	[65]
	1k–1o	4.500×10^1 [c]	1.000×10^2 [c]	[65]
	1m–1p	2.900×10^2 [c]	8.200×10^1 [c]	[65]
	2a–2c	3.740×10^3 [c]	6.900×10^2 [c]	[65]
	2a–2f	3.740×10^3 [c]	1.480×10^3 [c]	[65]
	2b–2d	1.380×10^3 [c]	3.800×10^2 [c]	[65]
	2b–2g	1.380×10^3 [c]	2.900×10^2 [c]	[65]
	2c–2f	6.900×10^2 [c]	1.480×10^3 [c]	[65]
	2d–2g	3.800×10^2 [c]	2.900×10^2 [c]	[65]
	2e–2i	1.540×10^3 [c]	1.070×10^3 [c]	[65]
	2j–2n	2.580×10^3 [c]	6.600×10^2 [c]	[65]
	2k–2o	1.500×10^2 [c]	1.200×10^2 [c]	[65]

Table S2. *Cont.*

Target	Compound	Activity		Reference
		Lower M_w	Higher M_w	
	2a–2i	5.114×10^{3} [c]	2.794×10^{3} [c]	[63]
	2b–2g	3.045×10^{4} [c]	2.337×10^{3} [c]	[63]
	2b–2l	3.045×10^{4} [c]	3.224×10^{3} [c]	[63]
	2c–2k	1.658×10^{4} [c]	3.986×10^{3} [c]	[63]
	2c–2m	1.658×10^{4} [c]	3.256×10^{3} [c]	[63]
	3a–3e	1.663×10^{3} [c]	3.229×10^{3} [c]	[63]
	3a–3k	1.663×10^{3} [c]	8.973×10^{3} [c]	[63]
	3b–3f	1.722×10^{3} [c]	6.039×10^{3} [c]	[63]
	3b–3l	1.722×10^{3} [c]	2.930×10^{3} [c]	[63]
	3c–3g	2.428×10^{3} [c]	1.520×10^{3} [c]	[63]
	3c–3m	2.428×10^{3} [c]	3.756×10^{4} [c]	[63]
	3d–3h	5.880×10^{2} [c]	4.509×10^{3} [c]	[63]
	3d–3n	5.880×10^{2} [c]	2.687×10^{4} [c]	[63]
	3a–3e	2.070×10^{5} [c]	1.900×10^{5} [c]	[64]
	3b–3f	2.880×10^{5} [c]	1.990×10^{5} [c]	[64]
	3c–3g	1.980×10^{6} [c]	2.510×10^{5} [c]	[64]
	3d–3h	1.370×10^{6} [c]	2.340×10^{5} [c]	[64]
	3i–3m	3.950×10^{4} [c]	5.550×10^{4} [c]	[64]
**hBuChE**	3j–3n	2.120×10^{5} [c]	1.580×10^{5} [c]	[64]
	3k–3o	8.430×10^{4} [c]	2.620×10^{5} [c]	[64]
	3l–3p	1.300×10^{5} [c]	1.760×10^{5} [c]	[64]
	1a–1c	8.930×10^{4} [c]	3.680×10^{4} [c]	[65]
	1a–1f	8.930×10^{4} [c]	7.100×10^{4} [c]	[65]
	1b–1d	3.150×10^{5} [c]	2.010×10^{4} [c]	[65]
	1b–1g	3.150×10^{5} [c]	8.200×10^{4} [c]	[65]
	1c–1f	3.680×10^{4} [c]	7.100×10^{4} [c]	[65]
	1d–1g	2.010×10^{4} [c]	8.200×10^{4} [c]	[65]
	1j–1n	4.160×10^{4} [c]	5.520×10^{4} [c]	[65]
	1k–1o	2.660×10^{4} [c]	8.460×10^{4} [c]	[65]
	2a–2c	2.520×10^{4} [c]	1.850×10^{4} [c]	[65]
	2a–2f	2.520×10^{4} [c]	3.200×10^{4} [c]	[65]
	2b–2d	4.070×10^{4} [c]	3.450×10^{1} [c]	[65]
	2b–2g	4.070×10^{4} [c]	1.170×10^{4} [c]	[65]
	2c–2f	1.850×10^{4} [c]	3.200×10^{4} [c]	[65]
	2d–2g	3.450×10^{4} [c]	1.170×10^{4} [c]	[65]
	2j–2n	4.220×10^{4} [c]	7.040×10^{4} [c]	[65]
	2k–2o	3.320×10^{4} [c]	4.830×10^{4} [c]	[65]

Table S2. *Cont.*

Target	Compound	Activity		Reference
		Lower M_W	Higher M_W	
	2–3	1.923×10^{4} [c]	4.310×10^{3} [c]	[61]
	2a–4a	1.200×10^{1} [c]	4.100×10^{1} [c]	[66]
	5a–6a	3.600 [c]	1.600×10^{-3} [c]	[66]
	5b–6b	2.700×10^{-1} [c]	8.900×10^{1} [c]	[66]
	Harmine–3a	1.690×10^{1} [b]	5.000 [b]	[62]
	Harmine–3b	1.690×10^{1} [b]	3.900 [b]	[62]
	Harmine–3c	1.690×10^{1} [b]	2.950×10^{1} [b]	[62]
	Harmine–3e	1.690×10^{1} [b]	3.600 [b]	[62]
	Harmine–3f	1.690×10^{1} [b]	4.300 [b]	[62]
	Harmine–3g	1.690×10^{1} [b]	1.260×10^{1} [b]	[62]
	Harmine–3h	1.690×10^{1} [b]	5.000 [b]	[62]
	3a–3b	5.000 [b]	3.900 [b]	[62]
	3a–3c	5.000 [b]	2.950×10^{1} [b]	[62]
	3a–3e	5.000 [b]	3.600 [b]	[62]
	3a–3f	5.000 [b]	4.300 [b]	[62]
	3a–3g	5.000 [b]	1.260×10^{1} [b]	[62]
	3a–3h	5.000 [b]	5.000 [b]	[62]
	3b–3c	3.900 [b]	2.950×10^{1} [b]	[62]
	3b–3e	3.900 [b]	3.600 [b]	[62]
**hMAO–A**	3b–3f	3.900 [b]	4.300 [b]	[62]
	3b–3g	3.900 [b]	1.260×10^{1} [b]	[62]
	3b–3h	3.900 [b]	5.000 [b]	[62]
	3c–3e	2.950×10^{1} [b]	3.600 [b]	[62]
	3c–3f	2.950×10^{1} [b]	4.300 [b]	[62]
	3c–3g	2.950×10^{1} [b]	1.260×10^{1} [b]	[62]
	3c–3h	2.950×10^{1} [b]	5.000 [b]	[62]
	3e–3f	3.600 [b]	4.300 [b]	[62]
	3e–3g	3.600 [b]	1.260×10^{1} [b]	[62]
	3e–3h	3.600 [b]	5.000 [b]	[62]
	3f–3g	4.300 [b]	1.260×10^{1} [b]	[62]
	3f–3h	4.300 [b]	5.000 [b]	[62]
	3g–3h	1.260×10^{1} [b]	5.000 [b]	[62]
	2–3	8.310 [c]	3.803×10^{1} [c]	[61]
	2a–4a	6.400 [c]	1.900×10^{2} [c]	[66]
	5a–6a	4.900 [c]	3.700 [c]	[66]
	5b–6b	1.000×10^{1} [c]	4.800 [c]	[66]
	4a–5a	4.780×10^{1} [b]	2.490×10^{1} [b]	[36]
	4b–5b	1.490×10^{2} [b]	4.490×10^{1} [b]	[36]
	Harmine–3f	1.208×10^{5} [b]	2.216×10^{2} [b]	[62]

Table S2. *Cont.*

Target	Compound	Activity		Reference
		Lower M_W	Higher M_W	
hACE	5–7	7.700 × 10¹ ᵇ	1.500 × 10² ᵇ	[44]
	5–8	$7.700 \times 10^{1\ b}$	$2.500 \times 10^{2\ b}$	[44]
	5–9	$7.700 \times 10^{1\ b}$	$4.000 \times 10^{1\ b}$	[44]
	5–10	$7.700 \times 10^{1\ b}$	$6.200 \times 10^{1\ b}$	[44]
	5–11	$7.700 \times 10^{1\ b}$	$4.700 \times 10^{1\ b}$	[44]
	5–13	$7.700 \times 10^{1\ b}$	$2.000 \times 10^{1\ b}$	[44]
	5–14	$7.700 \times 10^{1\ b}$	$1.800 \times 10^{1\ b}$	[44]
	7–8	$1.500 \times 10^{2\ b}$	$2.500 \times 10^{2\ b}$	[44]
	7–9	$1.500 \times 10^{2\ b}$	$4.000 \times 10^{1\ b}$	[44]
	7–10	$1.500 \times 10^{2\ b}$	$6.200 \times 10^{1\ b}$	[44]
	7–11	$1.500 \times 10^{2\ b}$	$4.700 \times 10^{1\ b}$	[44]
	7–13	$1.500 \times 10^{2\ b}$	$2.000 \times 10^{1\ b}$	[44]
	7–14	$1.500 \times 10^{2\ b}$	$1.800 \times 10^{1\ b}$	[44]
	8–9	$2.500 \times 10^{2\ b}$	$4.000 \times 10^{1\ b}$	[44]
	8–10	$2.500 \times 10^{2\ b}$	$6.200 \times 10^{1\ b}$	[44]
	8–11	$2.500 \times 10^{2\ b}$	$4.700 \times 10^{1\ b}$	[44]
	8–13	$2.500 \times 10^{2\ b}$	$2.000 \times 10^{1\ b}$	[44]
	8–14	$2.500 \times 10^{2\ b}$	$1.800 \times 10^{1\ b}$	[44]
	9–10	$4.000 \times 10^{1\ b}$	$6.200 \times 10^{1\ b}$	[44]
	9–11	$4.000 \times 10^{1\ b}$	$4.700 \times 10^{1\ b}$	[44]
	9–13	$4.000 \times 10^{1\ b}$	$2.000 \times 10^{1\ b}$	[44]
	9–14	$4.000 \times 10^{1\ b}$	$1.800 \times 10^{1\ b}$	[44]
	10–11	$6.200 \times 10^{1\ b}$	$4.700 \times 10^{1\ b}$	[44]
	10–13	$6.200 \times 10^{1\ b}$	$2.000 \times 10^{1\ b}$	[44]
	10–14	$6.200 \times 10^{1\ b}$	$1.800 \times 10^{1\ b}$	[44]
	11–13	$4.700 \times 10^{1\ b}$	$2.000 \times 10^{1\ b}$	[44]
	11–14	$4.700 \times 10^{1\ b}$	$1.800 \times 10^{1\ b}$	[44]
	13–14	$2.000 \times 10^{1\ b}$	$1.800 \times 10^{1\ b}$	[44]

Table S2. *Cont.*

Target	Compound	Activity		Reference
		Lower M_W	Higher M_W	
hNEP	5–7	$1.350 \times 10^{2\ b}$	$2.200 \times 10^{1\ b}$	[44]
	5–8	$1.350 \times 10^{2\ b}$	$1.400 \times 10^{2\ b}$	[44]
	5–9	$1.350 \times 10^{2\ b}$	$3.000^{\ b}$	[44]
	5–10	$1.350 \times 10^{2\ b}$	$4.800^{\ b}$	[44]
	5–11	$1.350 \times 10^{2\ b}$	$5.400^{\ b}$	[44]
	5–13	$1.350 \times 10^{2\ b}$	$1.800^{\ b}$	[44]
	5–14	$1.350 \times 10^{2\ b}$	$4.500^{\ b}$	[44]
	7–8	$2.200 \times 10^{1\ b}$	$1.400 \times 10^{2\ b}$	[44]
	7–9	$2.200 \times 10^{1\ b}$	$3.000^{\ b}$	[44]
	7–10	$2.200 \times 10^{1\ b}$	$4.800^{\ b}$	[44]
	7–11	$2.200 \times 10^{1\ b}$	$5.400^{\ b}$	[44]
	7–13	$2.200 \times 10^{1\ b}$	$1.800^{\ b}$	[44]
	7–14	$2.200 \times 10^{1\ b}$	$4.500^{\ b}$	[44]
	8–9	$1.400 \times 10^{2\ b}$	$3.000^{\ b}$	[44]
	8–10	$1.400 \times 10^{2\ b}$	$4.800^{\ b}$	[44]
	8–11	$1.400 \times 10^{2\ b}$	$5.400^{\ b}$	[44]
	8–13	$1.400 \times 10^{2\ b}$	$1.800^{\ b}$	[44]
	8–14	$1.400 \times 10^{2\ b}$	$4.500^{\ b}$	[44]
	9–10	$3.000^{\ b}$	$4.800^{\ b}$	[44]
	9–11	$3.000^{\ b}$	$5.400^{\ b}$	[44]
	9–13	$3.000^{\ b}$	$1.800^{\ b}$	[44]
	9–14	$3.000^{\ b}$	$4.500^{\ b}$	[44]
	10–11	$4.800^{\ b}$	$5.400^{\ b}$	[44]
	10–13	$4.800^{\ b}$	$1.800^{\ b}$	[44]
	10–14	$4.800^{\ b}$	$4.500^{\ b}$	[44]
	11–13	$5.400^{\ b}$	$1.800^{\ b}$	[44]
	11–14	$5.400^{\ b}$	$4.500^{\ b}$	[44]
	13–14	$1.800^{\ b}$	$4.500^{\ b}$	[44]
hECE	5–7	$4.150 \times 10^{3\ b}$	$1.560 \times 10^{3\ b}$	[44]
	5–9	$4.150 \times 10^{3\ b}$	$3.000 \times 10^{2\ b}$	[44]
	5–10	$4.150 \times 10^{3\ b}$	$2.200 \times 10^{2\ b}$	[44]
	5–11	$4.150 \times 10^{3\ b}$	$9.800 \times 10^{2\ b}$	[44]
	5–13	$4.150 \times 10^{3\ b}$	$6.000 \times 10^{2\ b}$	[44]
	5–14	$4.150 \times 10^{3\ b}$	$3.000 \times 10^{2\ b}$	[44]
	7–9	$1.560 \times 10^{3\ b}$	$3.000 \times 10^{2\ b}$	[44]
	7–10	$1.560 \times 10^{3\ b}$	$2.200 \times 10^{2\ b}$	[44]
	7–11	$1.560 \times 10^{3\ b}$	$9.800 \times 10^{2\ b}$	[44]
	7–13	$1.560 \times 10^{3\ b}$	$6.000 \times 10^{2\ b}$	[44]
	7–14	$1.560 \times 10^{3\ b}$	$3.000 \times 10^{2\ b}$	[44]
	9–10	$3.000 \times 10^{2\ b}$	$2.200 \times 10^{2\ b}$	[44]
	9–11	$3.000 \times 10^{2\ b}$	$9.800 \times 10^{2\ b}$	[44]
	9–13	$3.000 \times 10^{2\ b}$	$6.000 \times 10^{2\ b}$	[44]
	9–14	$3.000 \times 10^{2\ b}$	$3.000 \times 10^{2\ b}$	[44]
	10–11	$2.200 \times 10^{2\ b}$	$9.800 \times 10^{2\ b}$	[44]
	10–13	$2.200 \times 10^{2\ b}$	$6.000 \times 10^{2\ b}$	[44]
	10–14	$2.200 \times 10^{2\ b}$	$3.000 \times 10^{2\ b}$	[44]
	11–13	$9.800 \times 10^{2\ b}$	$6.000 \times 10^{2\ b}$	[44]
	11–14	$9.800 \times 10^{2\ b}$	$3.000 \times 10^{2\ b}$	[44]
	13–14	$6.000 \times 10^{2\ b}$	$3.000 \times 10^{2\ b}$	[44]

^{a} k_i ($M^{-1}\cdot min^{-1}$); ^{b} K_i (μM); ^{c} IC_{50} (nM).
